# Bioinformatics perspectives on transcriptomics: A comprehensive review of bulk and single‐cell RNA sequencing analyses

**DOI:** 10.1002/qub2.78

**Published:** 2025-01-06

**Authors:** Jorge A. Tzec‐Interián, Daianna González‐Padilla, Elsa B. Góngora‐Castillo

**Affiliations:** ^1^ Biotechnology Unit, Yucatan Scientific Research Center Merida Yucatan Mexico; ^2^ Center for Genomic Sciences, National Autonomous University of Mexico Cuernavaca Morelos Mexico; ^3^ CONAHCYT‐Biotechnology Unit, Yucatan Scientific Research Center Merida Yucatan Mexico

**Keywords:** bioinformatics tools, next generation sequencing, RNA‐seq, scRNA‐seq, transcriptome

## Abstract

The transcriptome, the complete set of RNA molecules within a cell, plays a critical role in regulating physiological processes. The advent of RNA sequencing (RNA‐seq) facilitated by Next Generation Sequencing (NGS) technologies, has revolutionized transcriptome research, providing unique insights into gene expression dynamics. This powerful strategy can be applied at both bulk tissue and single‐cell levels. Bulk RNA‐seq provides a gene expression profile within a tissue sample. Conversely, single‐cell RNA sequencing (scRNA‐seq) offers resolution at the cellular level, allowing the uncovering of cellular heterogeneity, identification of rare cell types, and distinction between distinct cell populations. As computational tools, machine learning techniques, and NGS sequencing platforms continue to evolve, the field of transcriptome research is poised for significant advancements. Therefore, to fully harness this potential, a comprehensive understanding of bulk RNA‐seq and scRNA‐seq technologies, including their advantages, limitations, and computational considerations, is crucial. This review provides a systematic comparison of the computational processes involved in both RNA‐seq and scRNA‐seq, highlighting their fundamental principles, applications, strengths, and limitations, while outlining future directions in transcriptome research.

**Abbreviations**
ATAC‐Seq: assay for transposase‐accessible chromatin using sequencing
BBKNN: batch balanced K nearest neighbors
BEER: batch effect estimation and removal
BLADE: batch learning on approximate deconvolution estimates
BUSCO: benchmarking universal single‐copy orthologs
CAM: cellular annotations using mapping
CIDR: clustering through imputation and dimensionality reduction
EPIC: ensemble of probabilistic inference for cellular composition
FaStaNMF: fast and stable non‐negative matrix factorization
GEO: gene expression omnibus
HISAT: hierarchical indexing for spliced alignment of transcripts
LIGER: linked inference of genomic experimental relationships
MAST: model‐based analysis of single‐cell transcriptomics
MMD‐ResNet: maximum mean discrepancy residual neural network
MuSiC: multi‐subject single‐cell deconvolution
PAGA: partition‐based graph abstraction
SC3: single‐cell consensus clustering
SCNN: single‐cell neural network
SCORPIUS: single‐cell ordering by probabilistic inference and unsupervised learning
scVAE: single‐cell variational autoencoder
SCVIS: single‐cell visualization
scziDesk: single‐cell zero‐inflated deep embedding for scalable clustering
Smart‐seq: switching mechanism at 5′ end of RNA transcript sequencing
STRT‐seq: single‐cell tagged reverse transcription sequencing
*t*‐SNE: *t*‐distributed stochastic neighbor embedding
UMAP: uniform manifold approximation and projection
VASC: variational autoencoder for single‐cell
WGCNA: weighted gene co‐expression network analysis

## 1 INTRODUCTION

The transcriptome is a dynamic and complex entity that plays a central role in cellular biology. The transcriptome comprises the complete set of RNA molecules synthesized by a cell, and it influences various cellular processes. The remarkable adaptability of the transcriptome allows cells to fine‐tune gene expression in response to internal cues, environmental stresses, and developmental signals. Understanding these transcriptome dynamics is key to gaining a deeper understanding of the fundamental biological processes that govern health and disease. The advent of RNA sequencing (RNA‐seq) facilitated by next‐generation sequencing (NGS) technologies has revolutionized our ability to explore and understand the transcriptome. RNA‐seq allows measuring the abundance of the RNA molecules, thereby providing a comprehensive picture of gene expression. Bioinformatics analyses applied to transcriptomic data enable the identification of differentially expressed genes (DEG), elucidate gene regulatory networks, and track changes in the transcriptome under different conditions. Over the past decade, the development of NGS platforms has significantly reduced sequencing costs while enhancing accuracy, allowing more comprehensive studies of gene expression, and the discovery of novel RNA species and viral pathogens, as recently experienced in the SARS‐CoV‐2 pandemic. These methodologies can be applied to both, the tissue level, known as bulk RNA‐seq, and the individual cell level, referred to as single‐cell RNA sequencing (scRNA‐seq). Bulk RNA‐seq analyzes the combined gene expression of heterogeneous cell populations, providing an average expression profile within a tissue sample, while scRNA‐seq examines gene expression patterns at the individual cell level, providing unparalleled resolution, revealing cellular heterogeneity, identifying rare cell types, and distinguishing between different cell populations [1, 2, 3, 4].

The information generated by these strategies and big data holds immense promise for developing new therapies and drugs. For instance, the rapid development of COVID‐19 vaccines showcased the potential of RNA technologies in addressing the global health crises [5, 6, 7, 8]. Beyond human health, these technologies also impact economically important sectors such as agriculture and animal husbandry. Research efforts have focused on identifying genes related to disease resistance, tolerance to pathogens and abiotic stresses, and nutrient content in plants and animals [9, 10, 11, 12].

Furthermore, the rise of machine learning and artificial intelligence tools in recent years has fueled a surge in RNA‐based medical products. When combined with these new computational tools, RNA technologies hold immense promise for accelerating the development of innovative therapeutic solutions, such as personalized vaccines, improved existing technology for managing seasonal infections, and enhanced preparedness for future pandemics [13].

As the capabilities of computational tools continue to advance and the reliability and efficiency of RNA‐seq platforms improve, the field of transcriptome research is ideally positioned to make significant advancements. However, the majority of existing reviews focus on describing a single technology, either bulk RNA‐seq or scRNA‐seq. This absence of comparative analysis limits a comprehensive understanding of the similarities and differences between the two methodologies, as well as the specific computational tools required for each. This review aims to address this gap in the literature by providing a systematic comparison of the computational processes involved in both RNA‐seq and scRNA‐seq, highlighting their fundamental principles, applications, strengths, and limitations, while outlining future directions in transcriptome research. By navigating the computational landscape of both methodologies, we aim to provide a user‐friendly guide to enable researchers to effectively choose the method that best fits their biological context. A comprehensive grasp of both bulk RNA‐seq (hereafter referred to as RNA‐seq) and scRNA‐seq is essential to guarantee the accuracy of biological inferences, and driving innovation in several fields ranging from human health to agriculture.

## 2 SAMPLE PREPARATION AND SEQUENCING FOR RNA SEQUENCING AND scRNA SEQUENCING

**FIGURE 1:**
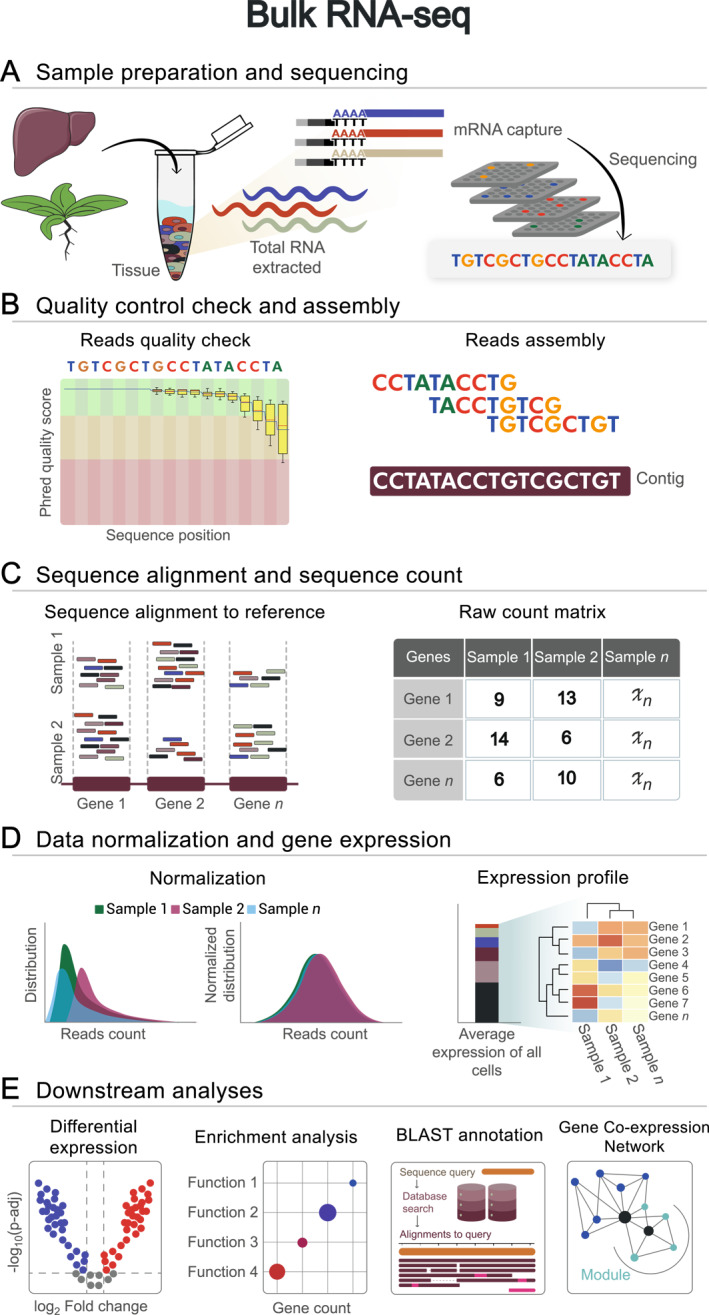
Bulk RNA sequencing workflow. (A) Sample preparation and sequencing, starting from RNA isolation from tissues to the sequencing process. Tissue source is represented by a liver and an Arabidopsis plant. (B) Quality control check, represented by Phred Quality Score plot, and reads assembly to obtain the contigs for *de novo* transcriptome assembly. (C) Alignment of sample sequences to genomic or transcriptomic references (each column represents the reads aligned to each gene) and mapped reads assigned, represented by the raw count matrix. (D) Data normalization per sample and average expression profile. (E) Examples of downstream analysis: volcano plot for differential expression, functions overrepresented in an enrichment analysis, query alignment representation in basic local alignment search tool annotation, and network construction and module detection in a gene co‐expression network analysis.

**FIGURE 2:**
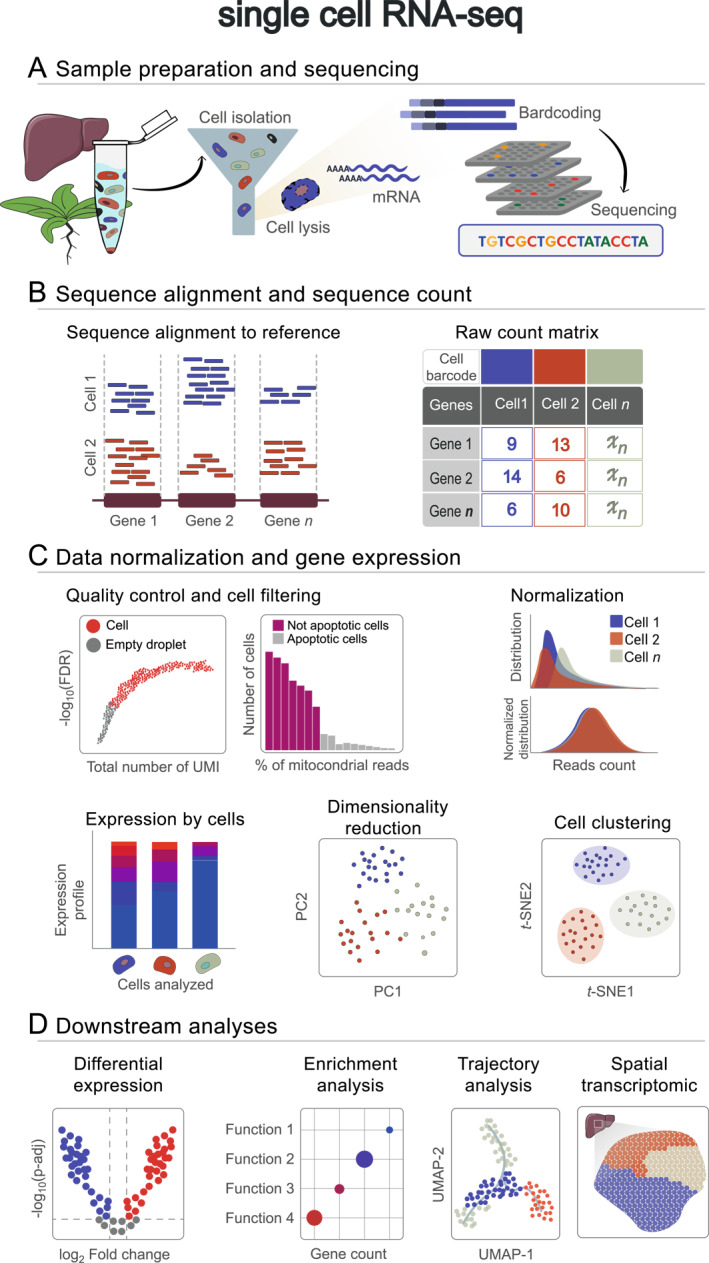
Single‐cell RNA sequencing workflow. (A) Sample preparation and sequencing. Liver and Arabidopsis plant tissues are represented. Various cell isolation methods are depicted by the funnel, followed by mRNA capture and sequencing. (B) Sequence alignment and counting. Reads aligned to a reference are counted per cell, generating a matrix representing reads/UMIs assigned per gene per cell. (C) Data normalization and gene expression. Quality control steps include UMI counts per cell determination (total number of UMIs vs. the false discovery rate on a logarithmic scale) and calculation of mitochondrial read percentages. Normalization per sample is depicted by histogram plots, while cell expression is visualized in a bar plot. Dimensionality reduction via principal component analysis aids in identifying novel cell types and expression changes. Cell clustering is represented by a *t*‐distributed stochastic neighbor embedding plot with different colors indicating cell clusters. (D) Downstream analysis. This step encompasses differential expression analysis shown in a volcano plot, enrichment analysis, trajectory analysis, and integration with other omics data such as spatial transcriptomics. UMIs, unique molecular identifiers.

The initial step in RNA‐seq and scRNA‐seq analyses is the definition and preparation of samples (Figures 1A and 2A). This is an important element of the experimental design in RNA‐seq studies, as it determines the scope of subsequent analyses. A primary objective is to delineate the experimental unit, defined as the individual subject to treatments or conditions for the purpose of assessing their impact. It is essential to consider all factors to be analyzed (categorical variables) and their levels in the experimental design. For example, He et al. conducted a comparative transcriptomic study on the response of *Machilus microcarpa* plants exposed to 25°C and −2.8°C, with temperature as the factor and the two magnitudes as the levels in 2022 [14]. When multiple experimental units receive the same treatment, they are considered replicates [15, 16, 17].

Comparisons can be made in a variety of configurations, including the contrast of two groups based on different phenotypes, multiple phenotypes or treatments, or the progression of stress or treatment over time (time course experiment). A crucial consideration at this stage is the determination of the number of replicates. It is typical to utilize three replicates as the standard in RNA‐seq analyses; however, this number may vary depending on the specific focus of the study. In exploratory analyses focused on establishing a hypothesis, the use of three replicates is sufficient [18]. Nevertheless, to monitor the expression of pertinent genes or to undertake a more comprehensive investigation into the response to a specific condition, the use of six or 12 replicates is advised [16]. The number of replicates has a direct impact on the estimation of DEG. The use of more replicates is highly efficient and helps to reduce biological variability and enhance precision [15, 16, 17, 19].

It is important to consider the randomization of experimental units to avoid the occurrence of batch effects. These effects refer to technical variations that arise when samples are processed in different batches and can lead to the introduction of systematic differences that may mask the true biological differences. A carefully devised experimental design, executed in accordance with the established protocol, is essential to generate reliable data and facilitate meaningful conclusions from RNA‐seq analyses [20].

Once the experimental unit, the number of replicates, the type of comparison to be performed, and the necessary considerations to ensure the reproducibility of the experiment, and to avoid contamination or artifacts of the process have been defined, the treatment is applied and the genetic material for sequencing is obtained. The process of generating cDNA libraries begins with the total RNA isolation directly from the tissue or sample of interest (Figure 1A). Diverse methods exist for this task, ranging from traditional Trizol‐based protocols [21, 22] to commercially available kits. It is significant to consider that the chosen method can subtly influence the abundance of certain transcripts, potentially introducing unwanted biases [23]. Following total RNA isolation, mRNA is selectively captured through poly(A) tails, serving as a template for cDNA synthesis. The resulting cDNA undergoes fragmentation and is amplified via polymerase chain reaction (PCR) [3] (Figure 1A). This fragmentation step is necessary for short‐read sequencing platforms like Illumina, as it facilitates efficient sequencing of shorter fragments. However, advancements in long‐read sequencing technologies like Oxford Nanopore bypass the need for fragmentation. This allows for direct sequencing of full‐length cDNAs, enabling the interrogation of alternative splicing isoforms and complete transcripts [24].

The decision between short‐ and long‐read sequencing is essential in genomics research, and RNA‐seq experiments depend on specific research goals. For studies primarily focused on quantifying and analyzing gene expression, short‐read platforms like Illumina dominate the landscape [25, 26, 27]. This preference stems from Illumina’s exceptional accuracy (<0.1% error rate) and substantial sequencing yield, reaching up to 40 billion short sequences on NovaSeq, according to the manufacturer’s specifications [28, 29]. However, when investigating alternative mRNA isoforms and epigenetic modifications, long‐read platforms like Oxford Nanopore and Pacific Biosciences (PacBio) single‐molecule real‐time (SMRT) technologies offer the unique capability to sequence entire mRNA transcripts [30, 31, 32]. Although an initial drawback of long‐read sequencing platforms compared to short reads was their high error rate of up to 20% [33], with the development and improvement of their technology, this rate has been reduced to values between 0.5% and 14%. It can be further improved to <0.02% when analyzing sequencing consensus [29, 34].

In the context of scRNA‐seq, workflow involves sample acquisition, single‐cell isolation, cell lysis, mRNA capture, reverse transcription, cDNA amplification, library construction, sequencing, and data analysis (Figure 2A). Unlike RNA‐seq, where replicates represent independent samples from the same condition, scRNA‐seq typically aims to compare cell populations between individuals under different conditions. The concept of biological replicates does not strictly apply since each cell is a unique entity, and technical variations during isolation and RNA capture can introduce significant inter‐cell variability. Due to the typically low yield of captured mRNA molecules, which results in numerous unexpressed genes and, consequently, a high number of zero counts, recent scRNA‐seq protocols aim to address these technical biases by using control sequences and applying robust statistical methods [35, 36, 37].

Several scRNA‐seq methods have been developed with varying levels of automation, throughput, and cost. These differences are reflected in the isolation of the cells, the transcript length in retrotranscription (full length or 3'/5' ends), the method for cDNA amplification (polymerase chain reaction or PCR or in vitro transcription or IVT), and the use of barcodes in library preparation (unique molecular identifiers or UMIs) [38, 39, 40, 41, 42, 43, 44]. Overall, these methods can be classified into two categories: full‐length sequencing methods and 3'/5' end‐sequencing methods (tag‐based methods). Full‐length sequencing methods include Quartz‐seq [45], Smart‐seq [46], and Smart‐seq2 [47] among others. Examples of 3' end‐based sequencing methods are CEL‐seq2 [48], Drop‐seq [49], inDrop [50], and 10x Genomics [51] and 5' end‐based sequencing is STRT‐seq [52]. Full‐length transcription scRNA‐seq methods are more useful for isoform analysis and allelic expression detection. However, due to their high cost, tag‐based scRNA‐seq technology remains more popular. At the time of writing this review, the most popular methods were droplet‐based microfluidics (microdroplets) [53], such as Drop‐seq, in Drop‐seq and 10x Genomics. These methods were preferred due to their low sample consumption, precise fluid control, and low operating costs. These methods incorporate UMIs into their protocol, enabling sample multiplexing and improving gene quantification and performance. Typically, cDNA amplification is necessary to generate sufficient material for high‐throughput sequencing. However, during cDNA amplification, base‐incorporation errors and biased amplification can occur, which may propagate through to the final sequenced library. To overcome biases associated with amplification, UMIs are introduced to barcode each mRNA molecule and screen out errors [40]. After library preparation, the libraries undergo high‐throughput sequencing.

## 3 QUALITY CONTROL AND TRANSCRIPTOME ASSEMBLY FOR RNA SEQUENCING

The resulting sequencing reads undergo quality control to ensure accuracy and reliability for downstream analyses. Quality assessment of RNA‐seq reads involves base trimming, low‐quality filtering, and removal of sequence contamination. Phred quality scores, which estimate the probability of base calling errors during sequencing, guide both base trimming and low‐quality filtering [54, 55] (Figure 1B). The identification of any remaining adapters or overrepresented sequences unrelated to the sample is essential for effective contamination removal [56, 57]. A variety of tools for the quality analysis of short reads are available, including FastQC [58], and FQStat [59]. Both tools provide a set of statistics to explore the quality, distribution, and other metrics of the reads obtained during sequencing accompanied by graphs for visualization of their reports. FastQC [58] is a widely used tool and currently supports the analysis of long reads derived from PacBio sequencing technology. As an alternative for quality analysis of long reads, Falco [60] is a viable option. It offers metrics similar to FastQC but with reduced analysis time.

Despite the generally low error rate in short‐read sequencing on the Illumina platform (<0.1%), low‐quality segments and adapter contamination can still occur. One of the most commonly utilized tools for filtering and trimming short reads is Trimmomatic [61]. Additionally, several tools are available that integrate quality analysis, filtering, and trimming for both short and long reads, including HTSQualC [62] and fastp [63]. Some tools, such as Porechop [64], were designed exclusively to find and remove adapters from Oxford Nanopore reads (Table 1). Thus, the selection of one or the other will depend on several factors, including the type of sequencing platform, the time allocated to this step, the quality identified, and the integration of one or more preprocessing steps.

**TABLE 1:** Frequently used tools for RNA‐seq transcriptome analysis and their main functions: From sequence preprocessing to common downstream analysis.

Step	Tool	Description
Quality control check	FQStat [59]	Short‐reads sequence quality analysis
	FastQC [58]	Short‐ and long‐reads sequence quality analysis
	Falco [60]	
	Porechop [64]	Filtering and removing adapters from Oxford Nanopore long‐read sequencing
	HTSQualC [62]	Quality check, filtering, and trimming short‐ and long‐read sequences
	fastp [63]	
	Trimmomatic [61]	Filtering and trimming short reads tool
Transcriptome assembly and QC	Trinity [65]	*De novo* assembler for short‐read sequences
	Oases [66]	
	SOAPdenovo‐trans [67]	
	rnaSPAdes [68]	
	TransLiG [69]	
	RNA‐Bloom [70]	
	RNA‐Bloom2 [71]	*De novo* assembler for long‐read sequences
	IDP‐denovo [72]	*De novo* assembler for short‐ and long‐read sequences
	SeqKit [73]	A suite of functions for sequence data operations, including format conversion, filtering, and manipulation of FASTA and FASTQ files. Also includes metrics generation for *de novo* assembly
	TransRate [74]	Quality assessment of *de novo* transcriptome assemblies in RNAseq analysis by evaluating contig and assembly performance metrics
	rnaQUAST [75]	Evaluates the quality of RNA‐seq transcriptome assemblies by comparing them to a reference genome and providing detailed metrics on accuracy and completeness
	BUSCO [76, 77]	Assesses the completeness of transcriptome assemblies by comparing them to a set of conserved, single‐copy genes across diverse species
	CD‐HIT [78]	Clusters and removes redundant sequences to reduce data complexity
Sequence alignment	Bowtie2 [79]	Aligner of short sequence reads to long reference sequences, facilitating the mapping of RNA‐seq reads
	STAR [80]	Maps short RNA‐seq reads to a reference genome with high precision and speed, enabling accurate analysis of gene expression and splice junctions
	HISAT2 [81]	Aligner of RNA‐seq reads to a reference genome, allowing analysis of gene expression and splice junctions
	StringTie [82]	Assembles and quantifies transcripts from RNA‐seq data, providing information on gene expression and isoform levels
Sequence count	HTSeq [83]	Assigns aligned reads to genomic features (genes or exons), enabling the quantification of gene expression
	featureCount from subread package [84]	Assigns aligned reads to genomic features, generating count matrices for downstream analyses
	Salmon [85]	Quantifies transcript abundance, providing estimation of gene expression levels without a reference genome
Data normalization and gene expression	DESeq2 [86]	An R package that performs differential gene expression analysis
	EdgeR [87]	
	Limma [88]	
Downstream analysis	ClusterProfiler [89]	Functional enrichment analysis
	pathfindR [90]	R package for functional enrichment analysis
	GSEApy [91]	Python package for functional enrichment analysis
	Weighted gene co‐expression network analysis (WGCNA) [92, 93]	R package that identifies gene co‐expression patterns to construct gene networks
	PyWGCNA [94]	A Python package for WGCNA

Upon completion of the quality assessment of raw short reads, RNA‐seq data can be assembled into a transcriptome using either *de novo* or reference‐guided approaches (Figure 1B). The *de novo* assembly reconstructs longer sequences from scratch, known as contigs, relying on *k*‐mers typically using *de Bruijn* graph algorithms [95]. Some of the most widely used *de novo* assemblers include Trinity [65], Oases [66], SOAPdenovo‐Trans [67], IDP‐denovo [72], rnaSPAdes [68], Trans‐ABySS [96], TransLiG [69], and RNA‐Bloom2 [71] (Table 1). One of the most popular *de novo* assemblers is Trinity [65], which has been referenced in over 14,000 publications since its initial release. It has been employed to study a diverse range of organisms, including prokaryotic organisms such as bacteria [97], eukaryotic organisms like plants [98] and reptiles such as Iberian ribbed newt [99], and numerous other species. Benchmarking studies have highlighted the features and advantages of Trinity and other assemblers. For example, Wang and Gribskov evaluated eight tools for *de novo* assembly of RNA‐Seq datasets from *Arabidopsis thaliana* in 2016 [100]. Their findings indicated that Trans‐ABySS demonstrated better performance in terms of gene coverage and full‐length transcript recovery. Conversely, Trinity and SOAPdenovo‐Trans exhibited the highest transcriptome quality when evaluated using *de novo* assembly metrics. In a recent study, Hölzer and Marz conducted a comparative analysis of 10 assemblers using 9 RNA‐Seq datasets derived from organisms across different kingdoms in 2019 [101]. The results indicated that Trinity and Trans‐ABySS achieved the highest overall metric scores and demonstrated proficiency in 95% assembled isoform rates for the majority of datasets. However, Trinity exhibited elevated memory consumption peaks during the initial assembly phases, and Trans‐ABySS demonstrated a strong performance in large datasets and human‐simulated data, but it exhibited a high duplication rate in most cases. With regard to the number of transcripts, Trinity achieved the highest values according to results described by Ahmadi et al. when conducting an analysis of seven assemblers in 2023 [102]. In general, comparative studies demonstrate that, although each tool has distinctive strengths, no single tool consistently outperforms the others across all. However, the assembly of all alternate transcripts from short reads remains a particularly challenging task due to the high complexity and diversity of alternative splicing events as well as the computational efficiency and accuracy required to address this challenge [103].

Long‐read sequencing has the advantage of avoiding the process of transcript assembly, which can lead to chimera formation and interfere with isoform analyses. Nevertheless, due to the error rate of long sequencing, long read assembly may be necessary depending on the research question. For instance, RNA‐Bloom2 can perform *de novo* assembly with long reads following a six‐step process including: (*i*) error correction, with or without the support of short reads using a de Bruijn plot; (*ii*) digital normalization; (*iii*) trimming and splitting of regions with shallow read depth; (*iv*) unitig formation; (*v*) polishing of unitigs; and (*vi*) deriving transcripts based on normalized read depth [71]. However, further investigation is necessary to assess its efficacy and ascertain its capacity for transcript assembly from long reads.

Once the assembly has been completed, the *de novo* assembled transcriptome must undergo quality control. Quality control typically involves redundancy reduction, statistical metrics, chimera detection, identification of universal orthologous genes, and contamination removal. Clustering highly similar sequences helps to reduce redundancy; tools such as CD‐HIT could be useful for clustering sequences based on identity and coverage thresholds [78]. Assembly statistical metrics, such as the number of contigs obtained, average contig length, and contig N50 and ExN50, provide valuable insights into the assembly quality. These metrics can be calculated using tools like SeqKit [73], TransRate [74], and rnaQUAST [75] (Table 1). It is important to interpret these metrics carefully. For instance, the N50 contig represents the contig length where half of the total assembled bases are contained in contigs of that size or larger, an excessively high N50 could potentially indicate the presence of chimeras [104, 105].

One of the most widely utilized tools for assessing the quality of *de novo* assembly is TransRate [74], as it enables the identification of incomplete assemblies, structural errors, and the presence of chimeras through the use of reads and assembly as input. As a reference‐free tool, it can be utilized in a diverse range of organisms and RNA types. In general, the ongoing development of novel algorithms for transcriptome assembly has allowed enhanced precision and versatility. However, the assembly of transcripts from polyploid organisms presents a significant challenge due to the elevated rates of fused or redundant transcripts. In this regard, the assembler TransLiG [69] has demonstrated the highest completeness and full‐length transcriptomes, particularly in autotetraploids, in comparison to Trinity [65] and SOAPdenovo‐Trans [67, 106]. In such instances, an assessment of the assembly using the TransRate [74] and the Benchmarking Universal Single‐Copy Orthologs (BUSCO) [76, 77] tools proved to be sufficient in evaluating the performance of TransLiG [69]. BUSCO tool is widely used for this purpose, the completeness score above 80% generally indicates a high‐quality assembly. BUSCO analysis categorizes the results into the following categories: complete single‐copy, complete duplicated, fragmented, and missing orthologs [77].

As previously stated, assemblers may encounter difficulties in defining transcript isoforms due to the generation of numerous repetitive sequences. Consequently, isoform redundancy reduction is often necessary when using the *de novo* assembled transcriptome as a reference for read mapping to mitigate overestimation of gene expression levels. This step could be achieved using representative isoforms [107, 108]. An additional evaluation of the assembly quality can be conducted through the identification of universal ortholog, which serves as a valuable proxy for estimating assembly completeness. This assessment entails a comparison of the predicted genes or proteins with those documented in OrthoDB [109].

This multifaceted quality control approach ensures the integrity and reliability of the *de novo* assembly for downstream analyses (Table 1).

## 4 SEQUENCE ALIGNMENT AND SEQUENCE COUNTS

Both scRNA‐seq and RNA‐seq involve mapping RNA reads to a reference genome or transcriptome (Figures 1C and 2B). However, the alignment of scRNA‐seq data presents unique challenges including the presence of low‐abundance transcripts and the inherent complexity of cellular heterogeneity. In contrast, the alignment of RNA‐seq data is generally straightforward due to the higher quality of the data. In both scenarios, the use of short reads is preferable, as they provide greater sequencer depth and enable the quantification of lowly‐expressed genes [110, 111]. As demonstrated in recent studies of endothelial cells in mice [112], and the role of homologous genes in regulatory networks in plant roots [113].

### 4.1 Sequence alignment and sequence counts for RNA‐seq

RNA‐seq alignment procedures usually require a genomic reference. However, when the organism’s genome sequence is unavailable, a *de novo* assembled transcriptome can serve as a reference, a common situation for non‐model organisms without sequenced genomes [107, 108].

Several tools have been developed to perform read alignments. Bowtie2 is a widely utilized aligner that facilitates alignment through the use of a genome index for efficient read localization and employing a secondary gap‐aware mapping process for enhanced speed, sensitivity, and accuracy, compared to gap‐unaware aligners [79]. Alternative, splice‐aware aligners like STAR, rely on seed alignments and clustering within specific reference regions to determine optimal alignments [80]. Both approaches produce SAM/BAM files that can be analyzed for read counting using tools such as HTseq [83] and featureCount [84] (Table 1). In the context of *de novo* assembled transcriptomes as reference, the number of mapped reads serves as a quality indicator for the *de novo* assembly, with higher mapping rates suggesting better assembly quality [114]. Tools like Salmon offer a one‐step approach for alignment and abundance quantification by quasi‐mapping reads to the *de novo* assembly. This tool accepts reads in FASTQ format or pre‐computed SAM/BAM alignments along with either a genomic reference or a *de novo* transcriptome assembly for quantification [85].

In contrast, utilizing a reference genome in RNA‐seq analysis provides a framework for the identification of novel transcripts or transcript isoforms. This process involves constructing a genome index for rapid alignment, utilizing a GTF or GFF file (an annotation file detailing known gene structures), and mapping reads using splice‐aware aligners, such as TopHat [115], STAR [80], HISAT/HISAT2 [81], Cufflinks [116], Scallop [117], and StringTie [82]. StringTie stands out for its versatility, as it is capable of handling both short and long reads (Table 1).

In general, comparative studies between different aligners have demonstrated that their impact on DEG identified is not significantly different when mapping to a reference genome. Nevertheless, tools that fully map reads against the transcriptome have been observed to produce results that are different from those produced by quasi‐mapping methods [118]. While the overall performance of aligners is comparable, metrics such as alignment rate and gene coverage can influence the selection of one aligner over another. For instance, in RNA‐seq datasets for the fungus *Erysiphe necator*, BWA has shown robust performance in both alignment rate and gene coverage [119]. In terms of obtaining the longest transcripts, HISAT2 and STAR demonstrated robust performance, with HISAT2 occasionally exhibiting higher speed than other aligners [120]. These characteristics are dependent on the complexity of the dataset and the selected alignment parameters.

### 4.2 Sequence alignment, sequence counts, and QC for scRNA‐seq

In scRNA‐seq, there are several aligners available to align to a genomic reference (Figure 2B). Examples of these tools include Cell Ranger, that employs the splicing‐aware aligner STAR [80]; STARsolo, is an aligner that was built directly within the STAR code [121, 122]; Alevin, is integrated with Salmon software for mapping and quantification, generating a cell‐by‐gene count matrix [118]; Alevin‐fry, incorporates features of safety, accuracy, and efficiency [123]; and Kallisto, employs a pseudo‐alignment approach to accelerate the alignment process [124] (Table 2).

**TABLE 2:** Tools for single cell RNA‐seq transcriptome analysis and their main functions.

Step	Tool	Function/description
Reads preprocessing	FastQC [58]	Quality check
	fastp [63]	Filtering the adapter sequence and removing the low‐quality reads
	UMI‐tools [125]	Cell barcodes UMI analysis
Alignment	STARsolo [121, 122]	Correcting errors and demultiplexing cell barcodes, mapping reads to the reference genome using the STAR, error correction and collapsing of UMIs, quantification of per‐cell gene expression, and quantification of other transcriptomic features
	Alevin [118]	Process droplet‐based single‐cell RNA sequencing data and performs cell barcode detection, read mapping, unique molecular identifier (UMI) deduplication, gene count estimation, and cell barcode whitelisting
	Alevin‐fry [123]	An improved version of alevin that incorporates faster processing and lower memory usage. It includes salmon for alignment and quantification
	Kallisto [124]	Performs a pseudo‐alignment of the reads to a reference
	HISAT2 [81]	Fast and accurate alignment tool used for aligning RNA sequencing reads to a reference genome
UMI counting and deduplication	scPipe [126]	An R package for barcode demultiplexing, transcript mapping, and quality control.
	CELseq2 [48]	A Python toolkit designed to produce the UMI count matrix from CEL‐Seq2 sequencing data
	zUMIs [127]	For processing RNA‐seq data with or without UMIs, efficiently collapsing UMIs and handling both known and random barcodes for exon and intron mapping reads, with input being fastq files and requiring a STAR index and GTF annotation file
	Scruff [128]	An R package that preprocesses CEL‐seq or CEL‐Seq2 data for efficiently demultiplexing, aligning, and counting reads mapped to genome features with UMI tag deduplication
	Kallisto bustools [129]	For preprocessing stages, including cell origin association, UMI‐based read collapsing, and gene/feature count generation to create a cell‐by‐gene matrix
Quality control: Filtering, dimensionality reduction, clustering, and data cleaning	UMI‐tools [125]	To process the UMIs aligned to each gene. or UMI deduplication, identifying and removing duplicate reads
	CIDR [130]	Clustering via imputation and dimensionality reduction for scRNA‐seq data
	SCNN [131]	An example of a neural networks (NN) method applied for dimensionality reduction and other analysis of single‐cell RNA‐seq data,
	RaceID3 [132]	A tool utilizing random forest to refine k‐means clustering results
	DESC [133]	Tool incorporating autoencoder techniques into their clustering methodologies
Gene expression and other analysis	DEsingle [134]	Differential expression analysis tool for scRNA‐seq.
	MAST [135]	Differential expression analysis tool for scRNA‐seq focuses on zero‐inflated single‐cell data.
	edgeR [87]	Differential expression analysis of RNA‐seq data
	DESeq2 [86]	Differential expression analysis of RNA‐seq data
	SINCERA [136]	Preprocessing, cell type identification, cell type‐specific gene signature, and driving force analysis from raw or normalized expression values
Trajectory analysis	Slingshot [137]	To model developmental trajectories in single‐cell RNA sequencing data
	PAGA [138]	A tool for generating graph‐like cell maps that maintain continuous and disconnected structures across various resolutions
	SCORPIUS [139]	A tool for reconstructing cell orderings without prior dynamic process information
	Seurat [140]	R package for data quality analysis and preprocessing, dimensionality reduction and clustering analysis, visualization, cell state differentiation (pseudotime), marker analysis, data integration, and other analysis
	SCANPY [141]	A Python and R package toolkit for preprocessing, visualization, clustering, trajectory inference, integrating datasets, differential expression, and spatial data analysis of large‐size datasets
		Applies normalization and performs simulations
	Monocle2 [142, 143]	An R package toolkit for clustering, classifying, and counting cells, identification of marker genes, differential expression analysis, and most commonly used for constructing single‐cell trajectories across pseudotime

*Note*: Some tools perform different tasks of the scRNA‐seq data analysis process; however, they were classified at the stage where they are most commonly used or as a suite toolkit.

A comparative study of these aligners using different 10 × human and mouse datasets, revealed contrasting performance, depending on the metric evaluated. Although Kallisto proved to be the fastest in terms of run times along with Alevin‐fry, it also demonstrated the highest transcriptome mapping rate. The evaluation of cell count and the mean number of genes per cell yielded comparable results for Cell Ranger and STARsolo. In addition to 10x Genomics data, STAR and Kallisto were evaluated for Drop‐seq and Fluidigm, with STAR identifying a greater number of genes and higher gene expression values. The advantages of using the STAR aligner are offset by its slower computation time and higher memory usage [121, 122].

If a reference genome is accessible, tools like StringTie [82] and Scallop2 [144] enable the analysis of novel isoform structures in specific cells by assembling transcripts from scRNA‐seq data. In instances where a reference genome is absent, *de novo* transcriptomes can be constructed from single‐cell data. RNA‐Bloom has exhibited superior performance in reconstructing the entire transcriptome of single cells using both reference‐guided and reference‐free methods [70]. Additionally, full‐length transcriptome sequencing serves as an alternative in the absence of a genome reference [145].

The inherent nature of scRNA‐seq data presents unique challenges due to high variability between cells, which arises from both RNA capture and stochastic transcription in cells. Biological factors that may influence single‐cell data include cell sizes, gene expression, and cell states. Therefore, stringent quality control measures are indispensable to ensure robust results and minimize the influence of technical and biological noise. This involves identifying and eliminating low‐quality data, such as doublets (two or more cells captured together), dead cells, and reads associated with low capture efficiency or uneven amplification. Common metrics for assessing the integrity and suitability of individual cells include gene counts, UMI counts, and proportion of mitochondrial and ribosomal genes. Gene counts help to identify excessively high or low values, potentially indicating doublets or poor RNA quality, respectively. UMI counts reflect overall RNA abundance in a cell, with low counts suggestive of dead cells or inadequate capture, and high indicating potential doublets. Elevated expression of mitochondrial and ribosomal genes indicates cell stress or death (Figure 2C). Additionally, evaluating metrics like random undetected genes (dropouts) as indicative of a high proportion of unexpressed genes in a cell, amplification bias, and experimental batch effects is crucial for accurately interpreting downstream analyses.

For UMI counting, demultiplexing, and UMI deduplication, useful tools include UMI‐tools [125], scPipe [126], CELseq2 [48], scruff [128], zUMIs [127] and Kallisto bustools [129] (Table 2). Although all of these tools are useful in preprocessing, they possess specific features that make them suitable for different situations. For instance, CELseq2 [48] and scruff [128] are appropriate to use with plate‐based CEL‐Seq2 protocols, whereas scPipe [126], zUMIs [127], and Kallisto bustools [129] can be used for both plate‐ and droplet‐based protocols. UMI‐tools are primarily utilized for the UMIs deduplication, identification, and removal of duplicate reads resulting from PCR amplification [125]. In contrast, scPipe and scruff are comprehensive end‐to‐end pipelines that include alignment, UMI counting, and quality control. zUMIs is compatible with a range of scRNA‐seq protocols [127], including those that do not utilize UMIs.

Benchmarking studies with scRNA‐seq data from human and mouse cell lines have demonstrated that, concerning plate‐based protocols, scruff, and zUMIs exhibited high memory usage and runtime requirements, whereas Kallisto bustools demonstrated the most optimal performance in these metrics. In the context of droplet‐based protocols, zUMIs and dropSeqPipe exhibited the highest memory and runtime requirements, while Kallisto bustools proved to be more efficient. With regard to the number of genes identified, scPipe, CELseq2, scruff, and zUMIs exhibited similar performance, whereas Kallisto bustools demonstrated a lower detection rate [146].

While no universally accepted threshold exists for each metric, established recommendations serve as a starting point for filtering. Common suggestions include excluding cells with gene count ≤100 or ≥6000 expressed genes, UMI count ≤200 UMIs, and mitochondrial gene proportion ≥10% [40, 147]. It should be noted that these values are subject to variation depending on the specific dataset, as well as the organism in the context of the study. In 2020, Andueza et al. established the endothelial cell dataset by selecting cells with gene counts greater than 200 and less than 7600, as well as cells with less than 10% mitochondrial content [112]. Conversely, Zhang et al. excluded genes expressed in fewer than three cells and selected cells with feature counts greater than 200 and less than 100,000 for the plant root cell dataset in 2023 [113].

In summary, the alignment and counting steps are common to both RNA‐seq and scRNA‐seq technologies. Both methodologies require mapping the reads to a reference genome, subsequently enabling the construction of a count matrix based on the alignment. However, the procedures for executing these shared steps diverge. In contrast to RNA‐seq, which assigns reads or features to genes on a per‐sample basis, scRNA‐seq performs the count on a per‐cell level. Quality control is required after read assignment in scRNA‐seq to remove or filter genes or cells, thus preventing the introduction of noise in normalization, expression, and other downstream analyses.

A comparison of the final outputs reveals some differences in characteristics. In RNA‐seq studies, the detection of approximately 15,000–30,000 genes in human datasets is typical [148]. In contrast, scRNA‐seq datasets, which are predominantly generated in the context of medical research, can include large‐scale datasets comprising over 25,000 genes across 10,000 cells. However, this frequently includes genes that provide no informative value, as they predominantly exhibit zero counts. Even after filtering, the data dimension may remain considerable, comprising over 10,000 genes. Therefore, alternative strategies, such as the selection of 1000–5000 highly variable genes (HVG), are employed for downstream analysis. (A more detailed discussion of this topic can be found in Section 5.2) [149].

## 5 DATA NORMALIZATION, GENE EXPRESSION, AND DOWNSTREAM ANALYSIS

RNA‐seq and scRNA‐seq differ significantly in their normalization techniques, gene expression analysis, and downstream analysis approaches. In RNA‐seq, normalization methods typically involve scaling read counts to account for differences in library size or distribution across samples, aiming to reduce technical biases. Conversely, scRNA‐seq normalization techniques focus on addressing challenges such as variability in sequencing depth and cell‐to‐cell variation. Gene expression analysis in RNA‐seq focuses on quantifying average expression levels across populations of cells, providing insights into collective gene expression patterns. In contrast, scRNA‐seq allows for the examination of gene expression profiles at the single‐cell level, enabling the identification of rare cell types and cellular heterogeneity. Downstream analysis in RNA‐seq often involves differential expression analysis (DEA), pathway enrichment studies, and gene clustering based on expression patterns for elucidating molecular mechanisms underlying biological processes. For scRNA‐seq, downstream analysis may include cell and gene clustering, Trajectory inference (TI), and cell‐type identification, shedding light on cellular dynamics and lineage relationships.

### 5.1 Data normalization, gene expression, differential expression, and functional annotation for RNA‐seq

In RNA‐seq analysis, after aligning the reads to a reference (transcriptome or genome), the resulting counts are used to construct an expression matrix containing several biological samples and replicates (Figure 1C). These raw count matrices then undergo normalization to reduce experimental and technical biases within the samples [15, 150] (Figure 1D). Normalization methods are typically categorized into two groups: (*i*) normalization based on library size and (*ii*) normalization based on distribution. Library size‐based normalization includes methods like Reads Per Kilobase Million (RPKM), Fragments Per Kilobase Million (FPKM), and Transcripts Per Kilobase Million (TPM). Distribution‐based normalization methods include Quantile or Median normalization and DESeq normalization [151]. Currently, numerous tools incorporate various normalization strategies. For instance, Cufflinks uses FPKM [116], Salmon generates values in TPM [85], and DESeq2 [86] calculates the ratio of each read count to the geometric mean of all read counts for that gene across all samples. The median of these ratios for a sample, known as the “size factor,” is then employed to scale that sample’s count data [86] (Table 1).

The selection of a normalization method depends on the nature and goals of the experiment, which largely determines the pipeline and the programs used, according to each method’s specifications and compatibility with the dataset. Normalization significantly impacts the recovery of DEG and is a critical step in RNA‐seq analysis. The efficacy of normalization methods has been extensively reviewed and discussed by several authors [150, 151, 152, 153]. Comparisons of different normalization methods have been conducted, and the optimal method may vary depending on the performance metric utilized. A comparison of trimmed mean of M‐value (TMM), DESeq, Quantile, RPKM, TPM, and Log2 with human RNA‐seq datasets revealed that TPM yielded the most favorable results, exhibiting a notable correlation between site and samples with gene expression. This correlation was effectively preserved when using TPM than other methods under evaluation. However, an alternative study that evaluated FPKM, TPM, Z‐score on TPM, and count normalization (DESeq2 and TMM methods) found that count normalization methods achieved better clustering of replicates from the same patient‐derived tumors dataset when performance was assessed through hierarchical clustering analysis. It is also important to note that there are numerous methods for dealing with unbalanced transcriptomic data, including biological scaling normalization and hidden Markov model (HMM) normalization. This illustrates the necessity for a comprehensive examination of the data and an evaluation of diverse normalization techniques to ensure a fair comparison between samples and to obtain more accurate findings and conclusions [150, 151, 152, 153].

Identifying DEGs is a fundamental aspect of RNA‐seq analysis. This process allows for the discovery of genes whose expression changes under contrasting conditions (Figure 1E) [15, 154]. Several powerful tools in R facilitate this analysis, including DESeq2 [86], edgeR [87], and Limma [88] (Table 1). Each method uses different normalization strategies and statistical tests to identify DEGs and determine adjusted *p*‐values to distinguish between up‐ and down‐regulated genes. For instance, DESeq2 employs the Wald test or likelihood ratio test (LRT), EdgeR uses quantile‐adjusted conditional maximum likelihood (qCML) or generalized linear models.

A comparative analysis of the efficacy of various methods and tools for identifying DEGs indicates that NOIseq (which does not rely on parametric assumptions) [155, 156], DESeq2, and limma + voom produce the most optimal individual outcomes. The specificities demonstrated were 95%, 95%, and 93%, respectively, while the true positive rates were 80%, 84%, and 81%. These values were benchmarked against qRT‐PCR expression values [15, 154]. Furthermore, DESeq, DESeq2, and edgeR demonstrate effective control of the false positive rate (FPR) [19]. Several benchmarking studies suggest that DESeq2 and limma + voom display superior performance compared to other methods [19, 154, 157]. The choice of normalization method has a significant impact on the outcome. Thus, increasing the number of replicates or combining multiple methods can improve the sensitivity of DEG detection [157].

Once DEGs have been identified, the next step is to elucidate their biological role. This is typically achieved through enrichment analysis, which involves the use of databases like Gene Ontology (GO) and KEGG pathways [158, 159, 160, 161]. GO provides experimentally‐supported gene annotations across three categories: molecular function, biological process, and cellular component. Similarly, the KEGG pathways database provides insights into metabolism and related cellular processes. Both databases are valuable resources for Gene Set Analysis (GSA), which identifies enriched functions or processes within a set of genes (Figure 1E). Common tools for GSA include ClusterProfiler [89], g:Profiler [162], pathfindR in R language [90], GSEApy in Python [91], as well as the Enrichment Map program [163] (Table 1).

The number of analytical tools available to analyze potential functions and biological processes of gene sets, whether differentially expressed or resulting from clustering analysis, contrasts with the availability of reference genomes for many organisms. For example, the GO database contains over 600,000 experimentally‐supported GO annotations, while KEGG’s GenomeNet includes genomic information for 10,095 organisms, divided into 1057 eukaryotes, 8601 bacteria, and 437 archaea. Furthermore, the KEGG pathway database contains a total of 1,197,618 maps [158, 159, 160, 161]. While GO and KEGG offer comprehensive data for functional annotation for many organisms, in several instances, the annotations remain incomplete or absent. This can result in the introduction of bias in the findings or the limitation of biological interpretation [164]. In such cases, strategies such as the Basic Local Alignment Search Tool (BLAST) [165] and Hidden Markov Models (HMM) [166], are useful for alignment‐based or model‐based identification, respectively. It is also essential to have access to curated databases such as NCBI, Pfam [167], and PANTHER [168] (see the website of ebi.ac.uk) to ensure accurate functional annotation (Figure 1E).

Clustering methods have been employed for a variety of purposes in bioinformatics analyses. The most prevalent techniques employed for the analysis of RNA‐seq data are hierarchical and *k*‐means clustering. These methods are utilized in the initial steps of analysis to identify outliers in comparative analyses of samples with or without normalized data. Furthermore, they are employed to identify expression patterns among subsets of normalized genes, such as DEGs [169, 170]. Recently, a clustering algorithm has been developed as a strategy for identifying DEGs [171]. One of the most well‐known applications of clustering methods is the identification of co‐expressed genes that share expression patterns. The gene co‐expression networks (GCNs) provide valuable information about the relationship between genes using clustering‐based algorithms to construct modules based on their similar expression patterns [172] (Figure 1E). A widely used software package for this purpose is the weighted gene co‐expression network analysis (WGCNA) in R [92], with PyWGCNA offering an alternative in Python [94] (Table 2). GCN analysis has been applied extensively in several research fields. For instance, Quan et al. employed WGCNA to identify key immune‐related genes in ovarian cancer in 2021 [93], and Zhang et al. successfully identified clusters of genes that exhibited co‐expression responses to salt stress in the green microalga *Chlamydomonas reinhardtii* in 2023 [173].

Integrating RNA‐seq data with other omic data types, such as genomics and metabolomics, can significantly expand its analytical scope and facilitate deeper exploration of regulatory processes. For example, integrating large‐scale transcriptomic datasets with metabolomic data increases the odds of elucidating metabolites biosynthesis pathways with therapeutic potential [174, 175]. Proteomics is another omics approach that has been frequently integrated with transcriptomics. By combining gene expression analysis with protein quantification using mass spectrometry, increases the potential for the discovery of novel proteins. For example, Qie et al. merged RNA‐seq transcriptomic analysis with proteomic data from macrophages in mouse tissues, revealing distinct profiles between resident and recruited macrophages and their potential implications in immune response in 2022 [176]. In addition, the integration of transcriptomics with epigenomics has facilitated the investigation of regulatory processes. For instance, the development and stress response of economically significant crops, such as Chinese cabbage (*Brassica rapa*) [177]. Another area of research is the study of microbial communities, with the aim of understanding gene activity using metatranscriptomics. A notable example of the potential of this approach to study trans‐kingdom interactions is the work of Destras et al., in which the bacterial, viral, and human compositions of nasopharyngeal samples were analyzed in 2024 [178].

In summary, RNA‐seq studies have become a fundamental tool for understanding biological phenomena at the expression level. The integration of RNA‐seq with other omics approaches has facilitated a deeper understanding of biological processes and has led to significant contributions to the field.

### 5.2 Data normalization, dimensional reduction, cell clustering, gene expression, and downstream analysis for scRNA‐seq

The bioinformatic tools developed for scRNA‐seq analysis usually integrate methods for batch effect correction for unwanted technical variations resulting from sample processing, dimensionality reduction, cell clustering to group similar gene expression profiles, and cell TI to identify differentiation paths of cells within a population (Figure 2C).

Although scRNA‐seq generates expression profiles for thousands of genes per cell, a large proportion of these genes, known as “housekeeping” genes, exhibit minimal variation across cells and mask biologically relevant signals. To overcome this challenge, high‐variable genes (HVGs) are utilized to identify cell‐to‐cell variation and to distinguish differences in mixed‐cell populations. Selecting high‐quality HVGs is a key step for downstream analysis [40]. HVG detection methods are often composed of two steps: normalization and analysis of variation. Owing to batch effects affecting the number of detected HVGs, normalization is an important step (Figure 2C). There are various methods for normalization, including scaling, regression‐based, and External RNA Control Consortium (ERCC)‐based methods. Scaling methods normalize scRNA‐seq data with zero counts and remove cell‐specific biases by scaling their expression levels using constant factors. While regression‐based methods, such as DESeq, link the variance and mean of the negative binomial distribution over the observed counts. ERCC‐based methods estimate mRNA contents per cell relying on control sequences with constant and known amounts added to each cell’s lysate [179]. Additionally, a commonly used method is to transform read counts using a logarithm with a pseudocount such as one. A variety of RNA‐seq normalization techniques have been extensively applied in scRNA‐seq, with counts per million (CPM) normalization being one of the most prevalent. CPM is a count depth scaling method that is based on the assumption that all cells in the dataset initially had the same number of mRNA molecules, with count depth variations arising only from sampling differences [180]. It is important to select the appropriate normalization method and know the limitations. For instance, the scaling methods cannot account for individual batch effects, whereas the regression‐based methods are sensitive to batch effects [181]. Popular tools include Scran [182] and Seurat [140]. Scran performs a normalization similar to DESeq; however, its calculations are based on multiple pools of cells, which are a subset of the dataset. To identify HVG, it uses locally weighted scatterplot smoothing (LOESS) with the mean‐variance relationship of log‐transformed expression values to estimate technical variance in each gene. While Seurat performs normalization with the relative expression multiplied by 10,000. It uses variance divided by mean (VDM), assigns the VDMs into 20 bins based on their expression means, and then normalizes using *z*‐scores. In contrast, normalization methods utilized in RNA‐seq, such as DESeq or TMM, have yielded disparate outcomes. While some have been successful, they have not effectively addressed one of the essential characteristics of scRNA‐seq datasets: the prevalence of zero counts. Among the normalization methods designed for scRNA‐seq, BASiCS, simple normalization, Linnorm, GRM, Scran, and SCnorm demonstrated effective performance when analyzing cell grouping with principal component analysis (PCA) [183].

The plethora of available normalization methods presents a significant challenge in selecting the most appropriate one. Therefore, it is recommended that the choice of normalization method be made with due consideration of the intended downstream analyses. For example, scaling over genes, as performed by Seurat, ensures that all genes are weighted equally in subsequent analyses. In contrast, data from full‐length protocols may be better analyzed with normalization methods that take gene length into account [180, 184]. Readers can refer to comparative studies like Lytal et al. in 2020 and Yip et al. in 2019 for evaluation of different normalization methods and tools for HVG discovery, respectively [183, 185].

The dimensionality of scRNA‐seq data is high due to the large number of genes obtained, which can pose a challenge in identifying cell groups. However, this dimensionality is typically of low rank due to two main factors: *i*) many genes are co‐expressed, and *ii*) many genes can be found with zero counts due to the dropout events (>90%), in a typical cell profile. Therefore, dimensionality reduction is necessary to project high‐dimensional data into low‐dimensional space to visualize the cluster structures and development TI [181, 186] (Figure 2C). Thus, cell clustering aims to understand the relationship between different cell types at a specific time. Clustering algorithms are useful for grouping cells with similar expression profiles and separating them from those of different cell types, allowing identification of distinct cell populations and characterization of their unique properties. In contrast, TI is focused on discovering the temporal transitions of cells over time. The algorithms infer trajectories that describe the differentiation of immature cells into specific cell types. These trajectories provide valuable insight into developmental processes, cellular responses to stimuli, and disease progression [187].

A plethora of dimension‐reduction methods have been developed [131, 181, 186, 188]. Out of these, commonly used methods are PCA, *t*‐distributed stochastic neighbor embedding (*t*‐SNE) algorithm, Uniform Manifold Approximation and Projection, deep learning models, etc. PCA is the most widely used approach to project the data into a low‐dimensional space to discover genes with the highest variance. Popular software packages implementing these methods include SC3 [189], which applies PCA to transform the distance matrices; however, PCA is limited in capturing nonlinear relationships between cells existent due to the presence of high levels of dropout and noise [190]. Seurat, as a toolkit, provides many dimension reduction methods such as PCA and *t*‐SNE [140]. CIDR applies principal coordinate analysis [130]. In addition to these, deep learning models, such as neural networks (NN) and variational auto‐encoders, have been emerging to offer efficient and scalable solutions. Examples of these include SCNN [131], SCVIS [191], and VASC, etc [192, 193].

To identify cell types, clustering methods are a powerful tool. These methods can be roughly classified into four categories: (*i*) *k*‐means clustering, (*ii*) hierarchical clustering, (*iii*) graph‐based clustering, and (*iv*) density‐based clustering [181]. The most popular approach is *k*‐means, which iteratively identifies a number of *k* cluster centers and assigns cells to the closest one [194]. One of the disadvantages of this method is that because *k*‐means clustering uses a greedy algorithm, it tends to identify global clusters, which results in poor identification of rare cell types. Examples of tools based on *k*‐means methods are SC3 [189] and RaceID3 [132]. SC3 integrates individual *k*‐means clustering results with different initial conditions and finds a consensus across multiple runs. RaceID3 uses Random Forest to reclassify the results of *k*‐means clustering and introduces a function to calculate the mean dispersion within a cluster to help determine the appropriate number. Another commonly used technique is hierarchical clustering, which can utilize two strategies: agglomerative clustering and divisive clustering. These strategies establish a hierarchical relationship between cells and genes, facilitating the discovery of rare cell types in small groups of cells. Examples of tools that integrate hierarchical clustering into its pipeline are CIDR [130] and pcaReduce [195]. CIDR integrates dimension reduction and hierarchical clustering to provide stable distance estimation between pairs of cells. While pcaReduce uses an agglomerative hierarchical clustering approach with PCA.

Graph‐based clustering has gained popularity for large‐scale data analysis due to the limitations of *k*‐means and hierarchical clustering methods. Currently, three types of algorithms are used: the clique algorithm, spectral clustering, and Louvain’s algorithm [196]. The Louvain algorithm is the most widely used for scRNA‐seq data, but an improvement called Leiden [197] has recently been implemented in tools such as SCANPY [141] and Seurat [140], and it has been recommended to obtain ideal clustering for annotating cells [180, 184] (Table 2). Another set of clustering methods gaining popularity relies on deep learning techniques, which can be classified into supervised and unsupervised learning approaches. Among unsupervised learning methods, autoencoders stand out as a type of artificial neural network designed to learn efficient data representations and extract meaningful features. DESC [133], scziDesk [198], and scVAE [199] are examples of software that utilize autoencoder techniques in their clustering methodologies.

After preprocessing and initial analysis (Figure 2A–C), downstream analysis becomes feasible. Typically, downstream analysis of scRNA‐seq data comprises DEA, Gene Set Enrichment Analysis (GSEA), and cell trajectory analysis. Detecting DEGs in scRNA‐seq data helps to identify potential biomarkers or signatures between different cell types. Several DEA methods are currently available [200, 201, 202], and interestingly, comparative bioinformatics studies suggest no significant differences between tools designed specifically for scRNA‐seq and those designed for RNA‐seq [201]. However, performing these analyses presents both biological and methodological challenges. Biological challenges are related to the absence of a variety of different biological scenarios that can make identifying outliers, environmental influences, and random errors difficult. In addition, the databases for gene annotation are often based on microarray and RNA‐seq data, which may limit the annotation accuracy at the cellular level. A major methodological challenge in scRNA‐seq data is the high abundance of zero counts. Distinguishing between true zeros of biological nature and zeros derived from drop‐out events (where highly expressed genes in one cell are missed in another) remains controversial, as dropouts significantly contribute to the number of zeros counts and can complicate data interpretation. Several tools are available to perform DEA and GSEA analysis, including MAST [135], Monocle2 [142, 143], SINCERA [136], and DEsingle [134]. Recently, tools such as iDEA [203] have integrated DEA and GSEA into a single process (Table 2).

TI, also known as pseudotime analysis, is one of the fundamental analyses in scRNA studies, enabling the computational modeling of cellular dynamic processes. Briefly, TI depicts a dynamic process as a directed graph, where different trajectories along the graph represent cellular lineages. Individual cells are then projected onto these lineages and the distance along each trajectory is called pseudotime, a relative measure of their developmental stage (Figure 2D). This approach allows the visualization of cell development as a tree structure and cell cycles as a loop. A wide range of software tools exist for TI analysis. Recent studies have compared 45 different methods using more than 300 datasets [204]. A key distinction between these methods lies in their ability to either fix the topology or detect various topology types. Currently, seven possible topology types have been defined, ranging from simple linear or branched structures to complex connected or disconnected graphs [204, 205, 206].

The choice of the TI method hinges on the expected topology of the trajectory in the data, as the performance of a method is highly dependent on the actual trajectory type being studied. To mitigate bias from specific algorithms or parameter configurations, it is recommended to confirm the inferred trajectory and its results by using multiple TI methods. Popular tools include PAGA [138], Slingshot [137] and SCORPIUS [139].

A comparison of the steps involved in normalization, expression analysis, and downstream analyses between RNA‐seq and scRNA‐seq reveals that many RNA‐seq methods have served as a foundation for scRNA‐seq. Over time, these methods have been refined, and new tools have been developed to better suit the specific characteristics and limitations of scRNA‐seq datasets. It is also noteworthy that while RNA‐seq has been applied across numerous fields, scRNA‐seq has been primarily developed for biomedical research, particularly for cancer studies. While its application in fields such as agriculture is increasing, further exploration is needed in these areas, as well as the extension of its use to different biological systems. Commercially important plants that have been analyzed using scRNA‐seq include *Zea mays* L. (maize) and *Oryza sativa* L. (rice), in addition to *Arabidopsis thaliana*, which serves as a model plant. Many of these studies have focused on root architecture and meristem composition [207, 208, 209].

Additionally, the majority of research has been conducted on datasets derived from eukaryotic cells, particularly on human and mouse cell lines. There have been very few studies conducted on insects, such as the research on the nervous system of *Drosophila larvae* [210], or on microbial communities, such as the analysis of *Bacillus subtilis* cells at different stages of growth to identify metabolic changes and lifestyle adaptations [211, 212].

## 6 INTEGRATING MULTIPLE DATASETS FOR TRANSCRIPTOMIC STUDIES

### 6.1 Batch effect correction

The exponential growth of public RNA‐seq and scRNA‐seq datasets in repositories like NCBI offers a wealth of valuable information [213]. However, utilizing this vast resource effectively presents significant challenges. Inconsistencies introduced by variations in platforms, laboratories, and protocols across studies can introduce biases into the data, known as batch effects. These batch effects can distort gene expression patterns, making it difficult to identify genuine biological signals. For instance, differences in sample preparation or sequencing techniques can introduce systematic biases that overshadow true changes in gene expression. To avoid erroneous conclusions about gene function, regulation, and overall cellular processes, proper methods for correcting batch effects are necessary. As the amount of information stored in databases continues to grow exponentially, the development of robust batch effect correction methods has become a top priority to ensure that researchers can harness the full potential of this vast amount of data.

For scRNA‐seq, several batch effect correction methods have been developed that can be categorized into five groups [214, 215]: (*i*) Anchor‐based methods rely on identifying pairs of cells across batches with similar expression patterns (anchors) to guide batch integration and removal of batch effects. The Mutual Nearest Neighbors (MNN) algorithm is typically used in these methods and has been implemented in tools like MNN‐Correct [216], BEER [217], Seurat [140], Scanorama [218], and scMerge [219] (Table 3). (*ii*) Graph‐based methods construct weighted graphs between and within batches, using community detection algorithms to identify shared cell populations. Conos [220], which performs pairwise batch comparisons, and BBKNN [221], which utilizes *k*‐nearest neighbors within each batch, are representative examples (Table 3). (*iii*) Anchor‐graph‐based methods utilize both anchors and graph representations for corrections. LIGER exemplifies this approach and relies on the assumption that differences between datasets are due to technical variations and not of biological origins [222]. (*iv*) Deep learning‐based methods harness the power of deep NN to learn complex patterns in the data. Examples include MMD‐ResNet, which assumes batch differences in data distribution [223], and scGen, which utilizes a trained variational autoencoderVAE model to learn the data distribution from a reference batch [224] (Table 3). (*v*) Model‐based methods rely on specific assumptions about the data distributions or cell type clusters. These methods include ComBat, which adopts an empirical Bayes framework assuming a normal distribution [225], and Limma uses a linear model with a batch effect term [88] (Table 3).

**TABLE 3:** Examples of different tools applied for batch corrections in data integration, and supervised and unsupervised methods for RNA‐seq deconvolution.

Approach	Method or tool	Description
Batch effect correction methods	Seurat [140]	Tools for batch effect correction with mutual nearest neighbors (MNN) algorithm for anchor‐based methods
	MNN‐correct [216]	
	Scanorama [218]	
	Conos [220]	Graph‐based methods for batch effect correction
	BBKNN [221]	
	LIGER [222]	Anchor‐graph‐based methods, utilize both anchors and graph representations for corrections
	MMD‐ResNet [223]	Deep learning‐based methods
	scGen [224]	
	ComBat [225]	Model‐based methods rely on specific assumptions about the data distributions or cell type clusters
	Limma [88]	
Supervised deconvolution methods	MuSIC [226]	Estimating cell type proportions from bulk sequencing using multi‐subject single‐cell data
	EPIC [227]	Estimating proportions of immune, stromal, endothelial, and cancer cells from bulk gene expression data using a reference gene expression profile
	BLADE [228]	Estimates cell type composition and gene expression profiles per cell type in scRNA‐seq data
	InteRD [229]	Integrate deconvolution results from multiple scRNA‐seq datasets, refining estimates from reference‐based deconvolution with additional biological priors
	CIBERSORTx [230]	Estimates the abundance of different cell types within a mixed cell population using gene expression data
Unsupervised deconvolution methods	FaStaNMF [231]	Performs gene expression analysis with non‐negative matrix factorization (NMF) to deconvolve mixed cell type samples, offering global stability and speed without prior knowledge of model components
	DECODER [232]	Framework for automated *de novo* deconvolution and single‐sample compartment weight estimation, enabling dynamic and biologically sound compartment derivation without ad hoc parameters
	CDSeq [233]	R Implementation of CDSeq, a method that simultaneously estimates cell‐type proportions and cell‐type‐specific expression profiles using only bulk RNA‐seq data from multiple samples

In the context of RNA‐seq studies, GCN is a powerful tool for predicting gene function and regulation. Gene co‐expression analyses require a large number of samples, which are often only available from public repositories. Therefore, it is essential to mitigate the impact of the batch effect. In a recent study, Vandenbon evaluated 50 strategies for correcting batch effects across a vast dataset comprising over 400 human and mouse studies in 2022. The findings indicated that the ComBat tools [225] and the limma removeBatchEffect function [88] enhanced the quality of the network by 45% [234]. Similarly, Soto‐Cardinault et al. demonstrated the importance of normalizing data and identifying outliers to correct batch effects in 2023 [235].

### 6.2 Deconvolution

RNA‐seq deconvolution is a computational technique that infers the proportions of cell types within a heterogeneous sample based on gene expression profiles, mainly utilizing scRNA‐seq data. This approach is particularly valuable for analyzing heterogeneous tissues or complex biological samples, where the isolation of individual cell types is challenging [4, 236, 237, 238, 239]. The primary goal is to estimate the relative abundance of each cell type present in the sample. This approach has been extensively applied in biomedicine, particularly to identify the different cell types present in cancerous or brain tumorous tissue [239]. For example, Newman et al. employed a deconvolution technique to infer cell‐type‐specific gene expression profiles in various tumor types, including melanoma, obviating the need for physical cell isolation in 2019 [230]. Likewise, deconvolution has been utilized on bulk tumor transcriptome data to identify the developmental trajectories most closely associated with the tumor sample for the classification of cancer origin [240]. Additionally, this approach has been employed to elucidate the heterogeneity of various cell types in osteoarthritis synovial tissues by generating in silico scRNA‐seq data from bulk RNA‐seq data [241].

Over 50 deconvolution techniques have been developed, with ongoing efforts to benchmark their efficiency. Most methods rely on a reference matrix, which stores gene expression patterns specific to each cell type. When applied to RNA‐seq data, deconvolution algorithms aim to find the optimal combination of reference profiles that best produces the observed gene expression data in the bulk sample. Existing statistical deconvolution approaches fall into two major groups: supervised and unsupervised methods [4, 226, 238, 239, 242]. Supervised methods require a labeled dataset, where RNA‐seq samples are accompanied by their corresponding cell type proportions. Traditionally, these proportions can be obtained through experimental isolation of pure cell populations or, more recently, by leveraging scRNA‐seq data itself as a reference. In this approach, a reference matrix is constructed using gene expression profiles from scRNA‐seq data. Supervised methods then utilize the labeled dataset and the reference matrix to “learn” how to predict cell type proportions from the observed RNA‐seq data. Common machine‐learning techniques employed for this purpose include regression and support vector machines. Examples of these are MuSiC [226], EPIC [227], BLADE [228], InteRD [229], and CIBERSORTx [230] (Table 3). The accuracy of supervised deconvolution is heavily influenced by the quality and representativeness of the reference cell type signatures relative to the bulk expression profiles. High‐quality reference data leads to more accurate results. However, these methods may not perform well when applied to novel or diverse samples that deviate significantly from the training data used to develop the model [236, 238, 239, 242].

In contrast, unsupervised methods do not require any prior information about the cell type composition of the sample. They rely solely on the gene expression data from the bulk samples themselves. While unsupervised methods also utilize reference gene expression profiles, these references are typically obtained from external sources, such as publicly available datasets or databases. The goal is to decompose the bulk gene expression data into a combination of reference signatures corresponding to different cell types. Techniques like Non‐Negative Matrix Factorization (NMF) are commonly used for this purpose [232, 236]. NMF algorithms factorize bulk gene expression data into cell type‐specific expression profiles and corresponding cell proportions, with constraints like “non‐negative” and “sum‐to‐one”. Examples of NMF‐based methods include FaStaNMF [231] and DECODER [232]. Other unsupervised approaches widely used are CDSeq [233] and CAM [236] (Table 3). Unsupervised methods offer greater flexibility as they can be applied to a wider range of samples, including those lacking labeled training data. However, their performance can be highly sensitive to the quality of the reference data and may not perform as well as supervised methods when dealing with complex mixtures or noisy data.

As previously stated, the majority of deconvolution techniques have been employed in biomedical research. Recently, there has been an expansion into the deconvolution of RNA‐seq data from the model plant *Arabidopsis thaliana* [243, 244]. In light of these recent studies, a new avenue of research has been opened, paving the way for further applications of these techniques with the aim of reusing and expanding the information available from RNA‐seq data across different organisms.

## 7 OPPORTUNITIES AND CHALLENGES IN TRANSCRIPTOMICS

### 7.1 Expanding the scope of RNA‐seq

RNA‐seq is a well‐established technology, boasts high‐throughput sequencing platforms and diverse data analysis tools. The ongoing evolution of this field brings both new challenges and opportunities. For example, RNA‐seq can be used to identify aberrant gene expression that leads to Mendelian diseases. However, to guarantee a meaningful outcome, ensure unbiased results and clinical reproducibility, it is essential to standardize and automate the RNA‐seq protocol and computational workflow [245, 246]. More recently, several studies have explored RNA‐seq applicability to other RNA molecules like small and long non‐coding RNAs [247, 248]. For example, circulating microRNAs are a promising biomarker for many human diseases, especially cancer. MicroRNAs are found in many human fluids where they are highly stable. Quantification of these molecules and identification of their levels using RNA‐seq will serve as a biomarker for early detection of cancer and other diseases, as well as possible therapeutic agents [249]. This highlights the need for technological refinement and tool development to facilitate the analysis of these molecules effectively.

Integration of RNA‐seq with other omics data (e.g., proteomics, metabolomics) is a growing area enabling a more comprehensive exploration of biological phenomena [250, 251]. For instance, integrating RNA‐Seq with metabolomics is being used in agriculture research to explore physiological and metabolic changes associated with plant disease and to elucidate its pathophysiology. Correlating gene expression data with the metabolites during plant‐pathogen interaction facilitates the obtention of promising results and novel perspectives on crop tolerance and adaptability, as well as the advancement of molecular breeding, that can be used in marker‐assisted selection for crop improvement. Furthermore, RNA‐seq has been a valuable tool in elucidating the transcriptional regulatory networks involved in the interaction between plants and beneficial microorganisms. This has facilitated the identification of the promoter effects of biofertilizers on plant growth and nutrient uptake, thereby enhancing crop yield and quality [252].

### 7.2 Single‐cell multi‐omics, challenges and future directions in scRNA‐seq

In contrast, scRNA‐seq faces challenges across different stages. Expanding its application to diverse tissue types and achieving efficient cell isolation are primary challenges. Most of the scRNA‐seq studies have focused on higher organisms like mice and humans [253]. Studies on unicellular eukaryotic microorganisms (e.g., *Chlamydomonas reinhardtii)* or prokaryotes (bacteria) are limited [254, 255, 256, 257]. The rigid cell wall in these organisms makes it difficult to obtain sufficient RNA after cell lysis, and the lack of polyadenylated tails in some RNA molecules hinders their capture during sequencing [258]. Technological advances in cell lysis and RNA capture are necessary to overcome these limitations and expand scRNA‐seq applications to a broader range of organisms. Low and biased RNA capture also poses technical challenges, ultimately impacting data analysis and study scope. These challenges translate to low sequencing depth, a high number of zero reads, and underestimates of gene expression. Statistical strategies considering the specific nature of scRNA‐seq data are needed to address these biases [44, 259].

Currently, scRNA‐seq reads are mainly aligned to existing reference genomes. Although there are tools for constructing transcriptomes *de novo*, having easily accessible reference genomes is essential for the broader use of scRNA‐seq in various organisms and tissues [70]. Moreover, identifying cell populations is a critical step in scRNA‐seq analysis. Although there are some organism‐ or tissue‐specific atlases, the absence of high‐quality cell atlases for many different organisms poses a challenge in understanding cell identity, function, development, and regulation [260]. Therefore, the absence of this type of information hampers the process of cell type annotation and the discovery of new cell populations [261, 262, 263].

The vast repertoire of bioinformatic analysis protocols available for scRNA‐seq requires standardization efforts given the complexity of the data (see the website of scrna‐tools) [264]. Benchmarking studies are crucial to determine their scope, suitability, and areas to improve for good practices in analyzing transcriptomic data.

Despite the challenges that lie ahead, the methodological and technological advances in scRNA‐seq have been a significant turning point, particularly in the field of human health, but also in other areas such as agriculture. The advent of single‐cell multi‐omics technologies has enabled the simultaneous acquisition of genomic, epigenomic, transcriptomic, proteomic, and other omics profiling modalities, facilitating a deeper understanding of biological mechanisms and genotype‐phenotype relationships. Single‐cell multi‐omics typically involves the combination of scRNA‐seq with another omics strategy, with the goal of elucidating the relationship between gene expression and phenotype heterogeneity. The initial multi‐omics methods integrated genome and transcriptome sequencing (G&T‐seq, DR‐seq, TARGET‐seq) [265, 266, 267]. However, understanding the complexity of different phenotypes based solely on information derived from the coupling between genomic and transcriptomic profiles does not address the question of how the same DNA can have different expression patterns in different cells. The integration of transcriptomic and epigenetic information is the most widely used multi‐omic method. These methods correlate DNA methylation with gene expression to derive single‐cell genome‐wide methylome and transcriptome sequencing (scMT‐seq) methods [268]. The latest developments in chromatin accessibility profiling have led to the creation of the assay for transposase‐accessible chromatin sequencing (ATAC‐seq) [269, 270, 271, 272, 273], which identifies regions of the genome with open chromatin and links them to gene transcription. These techniques have been further refined to capture both epigenomic and transcriptomic profiles, including the assay for single‐cell transcriptome and accessibility regions (ASTAR‐seq) [274].

The integration of the transcriptome and proteome is one of the most commonly used multi‐omics strategies. The cellular indexing of transcriptomes and epitopes by sequencing and RNA expression and protein sequencing assay (REAP‐seq) methods are the most effective ways to obtain proteome and transcriptome profiles [275, 276]. The most recent multi‐omics modalities link transcriptomic phenotypes with genetic perturbations using CRISPR‐Cas9. These technological developments include CRISP‐seq, Mosaic‐seq, and Perturb‐seq [277]. Integrating scRNA‐seq with other omics data, such as genomics or epigenomics, is promising but faces several challenges. The limitations of existing protocols and the lack of standardized tools for integration represent significant obstacles to the effective implementation of this powerful approach [253, 278].

The rapid development of scRNA‐seq has been fundamental in the discovery of new cell types and has allowed the creation of cell atlases in different species, including the human Atlas (see the website of humancellatlas) [279, 280], the mouse Atlas (see the website of bis.zju.edu.cn/MCA) [281], and the fly Atlas [282]. Therefore, understanding the spatial organization of cellular compartments is essential for the formation and development of organs and tissues, and thus for the multicellular function of organisms. This field of study is known as Spatial Transcriptomics (ST). Several technologies have been developed to understand the spatial information of a cell and were named the 2020 method of the year by Nature Methods to recognize their importance [283]. ST at single‐cell resolution can be achieved by combining isolation strategies used in scRNA‐seq with in situ hybridization techniques and image analysis. The ST strategies are mainly divided into (1) Next‐Generation Sequencing (NGS)‐based, encoding positional information onto transcripts before next‐generation sequencing, and (2) imaging‐based approaches, comprising in situ sequencing‐based methods and in situ hybridization‐based methods [284, 285, 286]. This process yields a spatial map of gene activity in different regions of a tissue, allowing the identification of where and how genes are expressed in specific contexts. This field of study is expanding rapidly, with new machine and deep learning algorithms being developed daily to locate different transcripts in the cell using gene expression and related measures. The latest innovations in this field include novoSpaRc [287], Tangram [288], Celltrek [289], CytoSPACE [290], cell2location [291], SPOTlight [292], and many more. This technology has had an unprecedented impact on human health. It has transformed our understanding of a range of systems, including brain development and function [293, 294, 295], the identification of genes associated with schizophrenia and autism [296], spermatogenesis [297], heart and intestine development [298, 299]. Additionally, in the plant field we can find developmental studies in *A*. *thaliana* [300], however, they are still in their early stages compared to human biology.

scRNA‐seq and ST technologies have greatly expanded our understanding of intricate cellular communication. Cells coordinate to perform functions as a multicellular organism. Cell–cell interactions (CCI), also known as cell–cell communication or cell signaling, are the means by which cells coordinate their actions. These interactions are crucial for many biological processes, including cell growth, cell division and differentiation, tissue or organ development, and disease progression. We can capture the gene expression profile of tens of thousands of genes, many of which are related to CCI, for thousands and potentially millions of cells. This offers a great opportunity to study ligand–receptor interaction. Higher levels of expression of these genes reflect a greater likelihood of CCI, so this is a promising avenue of research. A plethora of bioinformatics tools have recently been developed to model and analyze CCI between and within cells based on gene expression data obtained from spatial and non‐spatial single‐cell transcriptomic data. Some of these include celltalker [301], CellPhoneDB [302], CellChat [303], among others. These computational tools allow us to infer an approximate CCI landscape from scRNA‐seq data (or ST data), advancing our understanding of CCI mechanisms in different biological systems. Particularly in cancer studies both TS and CCI have allowed the identification of key mechanisms that can change the state of normal cellular functioning to altered functions, providing deeper biological insights into this disease and paving the way for new treatments [286, 304, 305].

### 7.3 Unlocking the potential of public data: Data sharing and reproducibility in RNA‐seq

The exponential growth in transcriptomics research has generated vast amounts of data stored in public repositories like Sequence Read Archive (SRA), Gene Expression Omnibus (GEO) or The European Nucleotide Archive (ENA). This wealth of data has enabled new approaches that rely on the analysis of very large collections of public data by investigators who were not involved in the original data collection. However, integrating datasets across different studies and experiments remains a challenge due to variations in sequencing platforms and potential discrepancies in data quality (batch effects). Robust statistical methods and strategies are needed to address these challenges and enable effective analysis of public sequencing data.

Furthermore, while the genomics community has a solid tradition of data sharing, there is a clear benefit to applying best practices in data sharing. This helps to improve the quality of public data and ensures its reproducibility. Data should be shared through specific repositories whenever possible, such as those mentioned above, or access‐controlled repositories such as Figshare and Zenodo. It is crucial to include metadata, such as experimental conditions, sample type, and sampling details, to guarantee the reproducibility of the data. The unfortunate reality is that the guidelines for properly storing this information are limited. MINSEQE, or Minimum Information about Sequencing Experiments [306], sets standards for describing the minimum metadata needed from RNA‐seq experiments. This aims to avoid ambiguous interpretations and facilitates the reproduction of results. Furthermore, there is a clear need for standardization of computational analyses and detailed sharing of methods to ensure the reproducibility and consistency of results. However, studies by Simoneau et al. in 2021, show that only 25% of RNA‐seq articles provide comprehensive details on the methodology used in these studies, highlighting a significant gap in reporting standards [307].

Similar to RNA‐seq, the abundance of public scRNA‐seq data offers exciting possibilities for further exploration [233]. However, strategies for integrating data from different sources are still under development.

The scientific community has a tremendous opportunity to unlock new biological insights by addressing data storage issues related to the lack of best practices and developing robust methods to harmonize data across different RNA‐seq and scRNA‐seq studies.

## 8 CONCLUSION

RNA‐seq and scRNA‐seq technologies have revolutionized transcriptomics, enabling deeper insights into the complex dynamics of gene expression across various biological contexts, from human health to agriculture. RNA‐seq offers a comprehensive overview of gene expression in tissues, and scRNA‐seq provides unprecedented resolution at the single‐cell level, revealing cellular heterogeneity and dynamics. While both strategies aim to expand our understanding of gene expression and its effects on cellular dynamics by sequencing, aligning, and quantifying sequences, their methods of analysis differ significantly. It is essential to understand the differences between RNA‐seq and scRNA‐seq, as well as their data analysis strategies, in order to select the one that best fits the biological context. Furthermore, the appropriate methods, bioinformatics tools, and pipelines must be employed to accurately interpret the results and overcome technical challenges. Understanding the strengths and limitations of each approach allows researchers to make more accurate biological inferences, avoid misinterpretations, and propose improvements to overcome technological and methodological limitations. The integration of machine and deep learning into RNA‐seq and scRNA‐seq analyses is particularly promising, offering powerful tools for identifying new biomarkers, understanding disease mechanisms, and developing personalized therapeutic strategies. Moreover, integrating transcriptomics with other omics data holds immense potential for unraveling complex biological systems. In summary, the synergy between the strengths of bulk and scRNA‐seq technologies, cutting‐edge bioinformatics, and innovative computational algorithms, will undoubtedly lead to a new era of revolutionary discoveries in diverse fields of biology and medicine.

## AUTHOR CONTRIBUTIONS

**Jorge A. Tzec‐Interián:** Conceptualization; investigation; visualization; writing—original draft preparation; writing—review and editing. **Daianna González‐Padilla:** Investigation; writing—original draft preparation. **Elsa B. Góngora‐Castillo:** Conceptualization; project administration; supervision; writing—original draft preparation; writing—review and editing.

## CONFLICT OF INTEREST STATEMENT

The authors Jorge Tzec‐Interián, Daianna González‐Padilla and Elsa B. Góngora‐Castillo declare that they have no conflict of interest or financial conflicts to disclose.

## ETHICS STATEMENT

This article does not contain any studies with human participants or animals performed by any of the authors.

## Data Availability

Data sharing not applicable to this article as no datasets were generated or analyzed during the current study.
